# Adherence to antidiabetic medication and factors associated with non-adherence among patients with type-2 diabetes mellitus in two regional hospitals in Cameroon

**DOI:** 10.1186/s12902-019-0360-9

**Published:** 2019-04-03

**Authors:** Leopold Ndemnge Aminde, Maxime Tindong, Calypse A. Ngwasiri, Jeannine A. Aminde, Tsi Njim, Azingala Ajua Fondong, Noah Fongwen Takah

**Affiliations:** 1 Faculty of Medicine, School of Public Health, The University of Queensland, Brisbane, QLD 4006 Australia; 2 Non-communicable diseases Unit, Clinical Research Education, Networking and Consultancy, Douala, Cameroon; 3 Faculty of Medicine, Université Libre de Bruxelles, Brussels, Belgium; 4 Bamendjou District Hospital, Bamendjou, Cameroon; 5 Etoug-Ebe Baptist Hospital Yaounde (EBHY), Yaounde, Cameroon; 6 Department of International Public Health, Liverpool School of Tropical Medicine, Pembroke Pl, Liverpool, L3 5QA UK; 7 Health and Human Development Research Group (2HD), Douala, Cameroon; 8 Faculty of Health Sciences, University of Buea, Buea, Cameroon; 9 Department of Clinical Research, Faculty of Infectious and Tropical Diseases, London School of Hygiene and Tropical Medicine, London, UK

**Keywords:** Cameroon, Medication adherence, Type 2 diabetes

## Abstract

**Background:**

Diabetes mellitus is a growing cause of disease burden globally. Its management is multifaceted, and adherence to pharmacotherapy is known to play a significant role in glycaemic control. Data on medication adherence among affected patients is unknown in Cameroon. In this study, the level of adherence and factors influencing non-adherence to antidiabetic medication among patients with type-2 diabetes was assessed.

**Methods:**

A hospital-based cross-sectional study among adult patients receiving care in the diabetic clinics of the Limbe and Bamenda Regional Hospitals in Cameroon was conducted. Medication adherence was assessed using the Medication Compliance Questionnaire (MCQ). Factors associated with non-adherence to medication were determined using basic and adjusted multivariable logistic regression models.

**Results:**

A total of 195 patients with type 2 diabetes were recruited. The prevalence of non-adherence to medication was 54.4% [95% confidence interval (CI): 47.1–61.5%]. In multivariable analysis, age > 60 years (aO.R. = 0.48, 95% CI: 0.25–0.94), alcohol consumption (aO.R. = 2.13, 95% CI: 1.10–4.14) and insulin alone therapy (aO.R. = 2.85, 95% CI: 1.01–8.08) were associated with non-adherence. Patients attributed their non-adherence to forgetfulness (55.6%), lack of finances (38.2%) and disappearance of symptoms (14.2%).

**Conclusions:**

Adherence to anti-diabetic medication is poor in this study with more than half of participants being non-adherent. Urgent interventions are required to tackle this problem in combined efforts to stem this looming diabetes epidemic.

## Background

Diabetes mellitus is undoubtedly one of the fastest groing public health problems worldwide. According to the International Diabetes Federation (IDF), there were 415 million people living with diabetes in 2015, with a projected 642 million by 2040 [1]. Diabetes as was previously known, is not a disease of the rich, given that about 77% of the global burden of diabetes is in the low and middle income countries (LMICs), also significantly affecting rural and low socioeconomic populations. Also, diabetes is not only a disease of the elderly as about 50% of the patients are aged between 40 and 59 years [2]. The LMICs are faced with the challenge of tackling the growing burden of diabetes (including other non-communicable diseases) as well as the existing large burden of communicable and nutritional diseases [3]. By 2015, diabetes was responsible for 5 million deaths worldwide, which was far greater than deaths due to HIV/AIDS, tuberculosis, and malaria combined [1]. In a recent systematic review, the prevalence of diabetes in Cameroon stood at 5.8% [4], and findings from the Global Burden of Disease (GBD) 2016 study revealed that diabetes mellitus accounted for over 132,000 disability adjusted life years (DALY) and about 4000 deaths in Cameroon [5]. These demonstrate that the burden of diabetes on the society is enormous in terms of morbidity and mortality, and by extension, a significant impact on the economy and healthcare systems [6].

The management of diabetes is multifaceted including lifestyle modification, and pharmacotherapy [7]. Nonadherence to treatment has been a major huddle in the management of diabetes by healthcare providers. Also, the efforts made to explain and improve on adherence of patients to their treatment are not always effective [8]. Adherence, according to Vrijens et al., is defined as the extent to which patients are able to follow the recommendations for prescribed treatments [9]. Nonadherence could occur at different stages of their treatment. These include not starting the treatment at all, decision not to fill their prescription in the pharmacy, taking the wrong dose, or discontinue the treatment earlier than the last date [8, 9]. Depending on the environment and type of treatment, the methods of assessing adherence to medication include electronic monitoring methods, pill counts, patients and caregiver reports [10].

There have been several studies which have explored medication adherence to antidiabetic medications with varying results. A hospital-based study in the United Arab Emirates reported a prevalence of adherence to antidiabetic medications to be 84% [11]; while similar studies in Ethiopia and Uganda obtained prevalence of 85.1 and 83.3% respectively [12, 13]. Conversely, studies in Switzerland and Botswana provided lower prevalence of 40 and 52% respectively [14, 15]. Amongst others, some factors found to be associated with non-adherence to antidiabetic medication include financial difficulties, forgetfulness, younger age, level of education, existing diabetes complications and difficulties in taking the medications alone [11, 14, 16, 17]. The impact and consequences of non-adherence to antidiabetic medications largely include: increased costs to families especially in most African countries where healthcare costs are borne via out of pocket expenditures, increased overall country healthcare costs, worsening and or increased morbidity, and death [18–20].

Despite compelling evidence on nonadherence to antidiabetic medications and its consequences elsewhere, there is paucity of information in patients with diabetes in Cameroon. Therefore, our study aimed to determine the prevalence and identify factors associated with nonadherence to antidiabetic medications among patients with type 2 diabetes mellitus in two regional hospitals of Cameroon.

## Methods

### Study design and setting

This was a hospital-based cross-sectional study conducted from August to September 2016 in two regional hospitals; the Bamenda Regional Hospital (BRH) and the Limbe Regional Hospital (LRH), in the Northwest and Southwest regions of Cameroon respectively. Both institutions are secondary level healthcare facilities and act as the main referral centres for the respective regions. The two hospitals have dedicated diabetic clinics which provide care to persons with diabetes.

### Sampling and study participants

A consecutive sampling method was used to recruit eligible participants to the study. Adult (> 18 years) participants with a confirmed physician diagnosis of type 2 diabetes and receiving treatment during the study period were included in the study. Participants were recruited from the out-patient diabetic clinics. Individuals were excluded if they had acute life-threatening conditions such as coma or mental impairment which may have limited their cognitive ability to participate.

### Assessment of non-adherence

The Medication Compliance Questionnaire (MCQ) which has been previously validated for assessing medication adherence [21] was used. This tool was developed using a combination of the adherence scales including the Morisky self-reporting scale [22] and the Hill-Bone Compliance to High Blood Pressure Therapy Scale [23]. Validity of the MCQ tool has previously been assessed with a reported Cronbach’s alpha value of 0.782, suggesting acceptable reliability of the tool [21]. This questionnaire has seven questions and assessed patients’ intentional and unintentional nonadherence to medication instructions including reasons for nonadherence. A 4-point Likert scale for each question was used in the data collection tools: The response “Never” was given a score of 4; “sometimes (one to four times per month)”, a score of 3; “Often (more than five times per month or more than two times per week)”, a score of 2; “Always”, a score of 1. A total score for each patient was calculated which could range from 7 (minimum) to 28 (maximum). Adherence was defined as a score of 27 or more while non-adherence was defined by a score less than 27. This cut-off is guided by scoring system applied in the Morisky Medication Adherence Scale [24], if participants had taken atleast 95% of prescribed doses. This approach has been used in previous published studies [21, 24] and thus helped in comparability of our findings.

### Other data collected

The adherence tool described above was embedded in a self-administered data collection form in English language. Besides the assessment of medication adherence, data on socio-demographic characteristics like age, sex and educational level was obtained. Data on comorbidities, duration of diabetes, and presence of diabetic complications, treatment or drug type and method of glycaemic control were also collected. Smoking was defined as any individual who reported smoking at the time of the study and alcohol consumption was similarly defined as anyone who reported consuming alcoholic beverage.

### Ethical considerations

This study received approval from the Ethics committee of the Regional Delegation of the Ministry of Public Health. The study objectives and aims were in simple terms explained to eligible participants and only those who provided their signed consent were included in the study. All patient confidentiality was maintained and the study adhered to the World Medical Association’s declaration of Helsinki [25].

### Data analysis

Data were entered in Microsoft Excel 2016, and analysed using Stata IC version 13 statistical package (Texas, USA). Frequencies and percentages were computed for categorical variables and group comparisons done using the chi-squared test (or Fisher’s exact test where appropriate). Basic logistic regression models were used to investigate factors associated with non-adherence to anti-diabetic medication. Candidate predictor variables which were significant in the basic models were included in the multivariable regression model. Statistical significance was set at *p*-value < 0.05.

## Results

### Sociodemographic characteristics

A total of 195 patients were included in this study. The mean age was 60.5 ± 13.6 years and 70.3% were women. One hundred (51.3%) participants were recruited from the BRH. Seventy-two participants (36.9%) had achieved at least secondary education.

A total of 98 participants (50.3%) had duration of diabetes more than 5 years, and the overall mean body mass index (BMI) was 28.8 ± 5.4 Kg/m^2^. The main comorbidities found were: hypertension (*n* = 122, 62.6%), chronic renal disease (*n* = 7, 3.6%), heart failure (n = 7, 3.6%) and stroke (*n* = 5, 2.6%). As concerns the antidiabetic medications, 80 (41.0%) participants used a single oral hypoglycaemic drug, and 59 (30.3%) used at least insulin therapy. One hundred and seventy-four (89.2%) participants used the fasting blood sugar test as method for glycaemic control.

### Prevalence and reasons for non-adherence to antidiabetic medication

Overall, 106 participants were non-adherent to antidiabetic medications with a prevalence of 54.4% (95% confidence interval [CI]: 47.1–61.5%). Forgetfulness (30.2%), lack of finances (17.4%), disappearance of symptoms (7.7%) and being too busy (7.7%) were some of the main reasons highlighted by participants for non-adherence to their medication. Tables 1 and 2 respectively, present the prevalence and reasons for non-adherence. Table 3 shows a summary of patient responses to the medication adherence questions.

**Table 1:** Prevalence of non-adherence to antidiabetic medications among patient with type 2 diabetes mellitus in Limbe and Bamenda Regional Hospitals, Cameroon, 2016

Variable	Adherent n (%)	Non-adherent n (%)	Total N (%)	*p*-value
Overall, n (%) [95% CI]	89 (45.6) [38.7–52.5]	106 (54.4) [47.1–61.5]		
Age (in years)				
≤ 60	37 (35.9)	66 (64.1)	103(52.8)	**0.01**
> 60	52 (56.5)	40 (43.5)	92 (47.2)	
Educational level (*n* = 194)				
Never	26 (66.7)	13 (33.3)	39 (20.1)	**0.03**
Primary	35 (42.2)	48 (57.8)	83 (42.8)	
Secondary	14 (35.9)	25 (64.1)	39 (20.1)	
High school/University	13 (39.4)	20 (60.6)	33 (17.0)	
Gender (*n* = 194)				
Male	23 (39.7)	35 (60.3)	58 (29.9)	0.30
Female	65 (47.5)	71 (52.2)	136 (70.1)	
Co-morbidity**				
Hypertension (*n* = 121)	58 (47.9)	63 (52.1)	121 (100)	0.35
Chronic renal disease (*n* = 7)	2 (28.6)	5 (71.4)	7 (100)	0.37*
Heart failure (*n* = 7)	0 (0.0)	7 (100)	7 (100)	0.01*
Stroke (*n* = 5)	3 (60)	2 (40)	5 (100)	0.51*
Duration of diabetes (in years) [*n* = 192]				
< 5 years	49 (51.6)	46 (48.4)	95 (49.5)	0.07*
6–10 years	14 (31.8)	30 (68.2)	44 (22.9)	
11–20 years	17 (38.6)	27 (61.4)	44 (22.9)	
> 20 years	6 (66.7)	3 (33.3)	9 (4.7)	
Number of diabetic complications (*n* = 194)				
None	40 (50.0)	40 (50.0)	80 (41.2)	0.54
1	35 (42.7)	47 (57.3)	82 (42.3)	
2 or more	13 (40.6)	19 (59.4)	32 (16.5)	
Alcohol consumption (*n* = 194)				
Yes	26 (34.7)	49 (65.3)	75 (38.7)	**0.02**
No	62 (52.1)	57 (47.9)	119 (61.3)	
Tobacco use (*n* = 194)				
Yes	1 (33.3)	2 (66.7)	3 (1.5)	0.67*
No	87 (45.5)	104 (54.5)	191 (98.5)	
Current antidiabetic medications (*n* = 192)				
Single OHA	43 (53.8)	37 (46.3)	80 (41.7)	0.06
Two OHA	26 (48.2)	28 (51.8)	54 (28.1)	
Insulin	7 (26.9)	19 (73.1)	26 (13.5)	
Insulin and OHA	11 (34.4)	21 (65.6)	32 (16.7)	
Method of glycaemic control (*n* = 178)				
FBS	82 (47.1)	92 (52.9)	174 (97.8)	0.41*
Glycated haemoglobin	0 (0.0)	2 (100)	2 (1.1)	
Both	1 (50.0)	1 (50.0)	2 (1.1)	
Hospital setting (*n* = 195)				
BRH	46 (46.0)	54 (54.0)	100 (51.3)	0.85
LRH	43 (45.3)	52 (54.7)	95 (48.7)	

*Fisher’s exact test, **multiple response, *BRH* Bamenda Regional Hospital, *LRH* Limbe Regional Hospital, *FBS* fasting blood sugar, *OHA* oral hypoglycaemic agent

**Table 2:** Reasons for non-adherence to antidiabetic medications

Reasons for non-adherence (*n* = 133)	n (%)
Forgetfulness	59 (44.4)
Lack of finances	34 (25.6)
Drug not effective	2 (1.5)
Side effect of drug	6 (4.4)
Multiple medication	2 (1.5)
Too busy	15 (11.3)
Disappearance of symptoms	15 (11.3)

**Table 3:** Patient responses to the Medication Compliance Questionnaire (MCQ)

Questions	Responses n (%)			
	Never	Sometimes	Often	Always
How often do you forget to take your medicine?	98 (50.3)	69 (35.4)	19 (9.7)	9 (4.6)
How often do you decide not to take your medicine?	143 (73.3)	32 (16.4)	13 (6.7)	7 (3.6)
How often do you miss taking your medicine because you feel better?	151 (77.4)	30 (15.4)	6 (3.1)	8 (4.1)
How often do you decide to take less of your medicine?	161 (82.6)	28 (14.4)	4 (2.0)	2 (1.0)
How often do you stop taking your medicine because you feel sick due to side effects of the medicine?	176 (90.3)	12 (6.1)	5 (2.6)	2 (1.0)
How often do you forget to bring along your medicine when you travel away from home?	143 (73.3)	34 (17.5)	12 (6.1)	6 (3.1)
How often do you not take your medicine because you run out of it at home?	120 (61.5)	53 (27.2)	20 (10.3)	2 (1.0)

According to the Likert scale, “Never” = 4, “Sometimes” = 3, “Often” = 2, “Always” = 1

### Factors associated with non-adherence to antidiabetic medication

In bivariate analysis, age, educational level, duration of diabetes, alcohol consumption and insulin therapy were significantly associated with non-adherence. In multivariable analysis, participants who were aged > 60 years (adjusted odds ratio (aO.R.) = 0.48; 95% CI: 0.25–0.94, *p* = 0.02), participants who consumed alcohol (aO.R. = 2.13; 1.10–4.14, *p* = 0.04) and participants on insulin therapy (aO.R. = 2.85; 1.01–8.08, *p* = 0.04) were more likely to be non-adherent to their antidiabetic medication (Table 4).

**Table 4:** Factors associated with non-adherence to antidiabetic medication among patients with type 2 diabetes in Limbe and Bamenda Regional Hospitals, Cameroon, 2016

Variable	OR (95% CI)	Adjusted OR (95% CI)	*p*-value
Age (in years)			
≤ 60			0.02*
> 60	**0.44 (0.25–0.78)**	**0.48 (0.25–0.94)**	
Educational level			
Never gone to school			
Primary	**2.74 (1.24–6.08)**	1.71 (0.71–4.10)	0.52*
Secondary	**3.57 (1.40–9.08)**	1.58 (0.54–4.62)	
High school /University	**3.08 (1.17–8.07)**	1.60 (0.49–5.20)	
Gender			
Female			0.81*
Male	1.39 (0.75–2.60)	1.07 (0.05–2.28)	
Body-Mass Index, BMI (kg/m^2^)	0.99 (0.97–1.01)		0.55
Co-morbidity			
Hypertension			0.35
No			
Yes	0.76 (0.42–1.36)		
Chronic renal disease			
No			0.35
Yes	2.13 (0.40–11.25)		
Stroke			0.51
No			
Yes	0.54 (0.09–3.34)		
Duration of diabetes (in years)			
< 5 years			0.22
6–10 years	2.28 (1.08–4.84)		
11–20 years	1.69 (0.82–3.50)		
> 20 years	0.53 (0.13–2.26)		
Alcohol consumption			
No			0.04*
Yes	**2.05 (1.13–3.72)**	**2.13 (1.10–4.14)**	
Tobacco use			
No			0.67
Yes	1.67 (0.15–18.76)		
Number of diabetic complications			
None			0.54
1	1.34 (0.72–2.49)		
2 or more	1.46 (0.64–3.35)		
Current antidiabetic medications			
Single OHA			
Two OHA	1.25 (0.63–2.50)	1.16 (0.56–2.42)	0.04*
Insulin alone	**3.15 (1.19–8.34)**	**2.85 (1.01–8.08)**	
Insulin and OHA	2.22 (0.95–5.20)	0.78 (0.76–4.71)	

*p-value on multivariable analysis, *OHA* oral hypoglycaemic agent, *BMI* body mass index, *ref.* reference, *OR* odds ratio, *CI* confidence interval

## Discussion

In this cross-sectional study from two regional hospitals in Cameroon, not up to half of the patients attending diabetic clinics were adherent to their medication. This poor adherence to antidiabetic medications was largely driven by younger age, placement on insulin therapy and alcohol consumption. Forgetfulness, lack of finances, disappearance of symptoms and being too busy were the most frequent reasons given by participants as reasons for their non-adherence to medication.

More than half (54.4%) of our study participants were non-adherent to their diabetic medication. While this might imply a lack of attention people with diabetes give to their health, it may also reflect limitations in the diabetes care model or services in the study hospitals, with likely pint-sized or no patient counselling on the importance of strict adherence to their medication. Moreover, the reported prevalence of nonadherence is likely to be conservative as this was based on patient recall and self-reports which usually overestimate patient adherence levels. An almost similar result was obtained in Malaysia, where Ahmad et al. [21] showed that 53% of their respondents were non-adherent to medication. Another study conducted by Abebe et al. in Ethiopia showed a prevalence of 54.1% [26]. However, much lower rates of non-adherence have been seen in Uganda [13], Nigeria [27] and Palestine [28] reporting rates of 16.7, 27.5 and 42% respectively. This difference in adherence levels could be attributed to variations in the health care services, socio-economic status and metrics used for assessment of adherence across the study settings.

In multipredictor analysis, patients aged more than 60 years had a 52% significantly lower odds of being non-adherent to their medication compared to those less than 60 years. This is in agreement with studies elsewhere [29] which showed that non-adherence to medication is common in younger patients, generally attributed to challenges with accepting new diagnoses [30], limited disease knowledge, fear of side effects and burden of regimens [31]. Older patients with longer duration of disease are believed to be more aware about the disease and the importance of glycaemic control to prevent complications and also receive family support for managing their diabetes [12].

Patients on insulin therapy alone were twice as likely to be non-adherent compared to participants on oral hypoglycaemic agents (OHA). The common route of routine administration of insulin is via subcutaneous injections [32]. As such, fear and pain from the discomfort of these needle pricks could possibly deter patients from taking their medication, in part explaining their non-adherence. Also, insulin is much less available compared to OHAs and access remains poor in many regions of the world, thereby placing needing patients at risk of diabetes-related complications and death [13]. Moreover, affordability remains another challenge as insulin prices in private pharmacies are fairly high and vary considerably [33]. This would imply that many patients placed on insulin but who can’t afford are likely to go without treatment till they are able to purchase their medications or the next scheduled visit with their health care provider.

A two-fold increase in non-adherence was associated with alcohol consumption. Admittedly, alcohol consumption is associated with disadvantageous health behaviours, and previous studies have shown that alcohol use has an inverse relationship with frequency of patient hospital visits [34]. Our result is somewhat similar to those of Ahmed and colleagues who found that alcohol use was associated with poor adherence to diabetes self-care practices [35].

Among patient reasons for non-adherence to their medication, close to a third mentioned forgetfulness while one-sixth of patients indicated it was due to lack of financial resources for regular medication purchase. Inability to afford medication has been shown to be a very common reason for poor adherence [36]. In a nationally representative French study, after adjusting for potential confounders, Tiv and colleagues similarly identified financial constraint as a determinant for poor medication adherence among patients with type 2 diabetes [16]. Drug costs and affordability are recognized challenges to controlling chronic disease especially in low income settings like ours. Jingi et al. previously evaluated the costs and affordability of essential medicines for cardiovascular disease and diabetes care which they found to be largely unaffordable in the West region of Cameroon [37].

Forgetfulness has consistently been identified in a number of studies as a reason for non-adherence to medication [13, 21]. This may likely be due to the fact that patients do not receive adequate health education or lack appropriate family support. To address patient forgetfulness, there is need for more regular follow-up visits, counselling sessions involving a family member and even peer group campaigns [38]. Task-shifting via nurse-led approaches and community health workers for home visits on health education are likely to significantly improve adherence to medication, improve glycaemic control and overall health outcomes. Intervention studies using mobile technology for sending motivational message reminders have demonstrated improved medication adherence among people with HIV [39]. Policy makers and diabetes care providers could glean from such successful endeavours to improve medication adherence among patients with diabetes and other chronic diseases. Overall, Jimmy et al. suggest that identification of individual patient barriers to medication adherence and adaptation of suitable techniques may lead to better drug adherence [40].

This study had some limitations. First, assessment of medication adherence was based on self-reports which is liable to recall bias and may overestimate patient adherence status, when compared to other objective methods such as pill counts, prescription claim or biological assays [10]. As such it is highly likely that our report of non-adherence is a conservative estimate. Secondly, using a cross-sectional study design, our study is limited in establishing temporality and drawing on causal inferences, but only provides data on associations. In addition, the use of a convenience consecutive sampling may have led to selection bias as not all type 2 diabetes patients in the out-patient departments will have had follow-up visits during the recruitment process. As a result, interpretation of our findings in terms of generalizability should be done with caution. Furthermore, the small sample size in this study explained by the low turnout of patients in the outpatient diabetes clinics of the study centres highlights the need for larger studies to assess retention in care of diabetes patients in Cameroon.

Despite these drawbacks, our study has some merits, as a previously validated [21] medication adherence tool was used. This is among the few efforts in Africa, and to the best of our knowledge, the first study in Cameroon to provide evidence on antidiabetic medication adherence as well as the factors influencing non-adherence among patients with type 2 diabetes. These findings will be handy for government and policy makers as they design strategies for improving diabetes control in Cameroon.

## Conclusions

Our findings show that over half of the patients with type 2 diabetes receiving care in the two study hospitals were non-adherent to their anti-diabetic medication. Non-adherence was associated with being young, alcohol consumption and insulin only therapy. Key reasons for non-adherence included forgetfulness and lack of financial resources to obtain medication. Interventions to improve adherence to anti-diabetic pharmacotherapy and subsequent attainment of satisfactory glycaemic control, should include aggressive counselling and health education with a focus on younger patients recently diagnosed, those on insulin therapy and the control of unhealthy behaviours. While governments should consider subsidizing costs to make treatment largely affordable and available, population interventions targeting behavioural risk factors should be implemented and or intensified for primary prevention in the wider population.

## Abbreviations

aO.R.: Adjusted odds ratio
BMI: Body mass index
BRH: Bamenda Regional Hospital
DALY: Disability adjusted life years
FBS: Fasting blood sugar
GBD: Global Burden of Disease
IDF: International Diabetes Federation
LRH: Limbe Regional Hospital
MCQ: Medication Compliance Questionnaire
OHA: Oral hypoglycaemic agent
